# Bioactive Natural Compounds and Antioxidant Activity of Essential Oils from Spice Plants: New Findings and Potential Applications

**DOI:** 10.3390/biom10070988

**Published:** 2020-07-01

**Authors:** Lidiane Diniz do Nascimento, Angelo Antônio Barbosa de Moraes, Kauê Santana da Costa, João Marcos Pereira Galúcio, Paulo Sérgio Taube, Cristiane Maria Leal Costa, Jorddy Neves Cruz, Eloisa Helena de Aguiar Andrade, Lênio José Guerreiro de Faria

**Affiliations:** 1 Programa de Pós-graduação em Engenharia de Recursos Naturais da Amazônia, Instituto de Tecnologia, Universidade Federal do Pará, Belém 66075110, Brazil; leniojgfaria@gmail.com; 2 Laboratório Adolpho Ducke, Coordenação de Botânica, Museu Paraense Emílio Goeldi, Belém 66077-830, Brazil; angeloquimica17@gmail.com (A.A.B.d.M.); jorddynevescruz@gmail.com (J.N.C.); eloisa@museu-goeldi.br (E.H.d.A.A.); 3 Instituto de Biodiversidade, Universidade Federal do Oeste do Pará, Vera Paz Street, w/n Salé, Santarém 68040-255, Brazil; jmpgalucio@gmail.com (J.M.P.G.); pstjunior@yahoo.com.br (P.S.T.); 4 Programa de Pós-graduação em Engenharia Química, Instituto de Tecnologia, Universidade Federal do Pará, Belém 66075110, Brazil; cristianemlcosta@gmail.com

**Keywords:** bioactive compounds, bioactivity, Lamiaceae, Piperaceae, Lauraceae, radical scavenging activity

## Abstract

Spice plants have a great influence on world history. For centuries, different civilizations have used them to condiment the foods of kings and nobles and applied them as embalming preservatives, perfumes, cosmetics, and medicines in different regions of the world. In general, these plants have formed the basis of traditional medicine and some of their derived substances have been utilized to treat different human diseases. Essential oils (EOs) obtained from these plants have been also used as therapeutic agents and have shown supportive uses in remedial practices. The discovery and development of bioactive compounds from these natural products, based on their traditional uses, play an important role in developing the scientific evidence of their potential pharmaceutical, cosmetic, and food applications. In the present review, using recent studies, we exhibit a general overview of the main aspects related to the importance of spice plants widely used in traditional medicine: *Cinnamomum zeylanicum* (true cinnamon), *Mentha piperita* (peppermint), *Ocimum basilicum* (basil), *Origanum vulgare* (oregano), *Piper nigrum* (black pepper), *Rosmarinus officinalis* (rosemary), and *Thymus vulgaris* (thyme); and we discuss new findings of the bioactive compounds obtained from their EOs, their potential applications, as well as their molecular mechanisms of action, focusing on their antioxidant activity. We also exhibit the main in vitro methods applied to determine the antioxidant activities of these natural products.

## 1. Historical Importance and Traditional Uses of Spice Plants

Spice plants have a great influence on world history. For centuries, they have been used to condiment the foods of kings and nobles and as medicines to treat several human diseases. Furthermore, they were used as valuable items of exchange and trade, and as a monetary source. Their applications as components of perfumes, cosmetics, incenses, and embalming preservatives have been reported in different regions of the world, from China to Ancient Rome [1,2,3,4].

Seeds, gums, leaves, and roots from these plants with a pleasant odor and taste were used as a condiment by ancient civilizations. Smells and flavors were a basic source of information and played an important role in human life since the earliest times. The Indians used spices and herbs, such as black pepper and cinnamon for gastronomy and medicine. The ancient Egyptians used fragrances and incense for religious purposes, and the Greeks discovered that the flavors of some plants had invigorating and refreshing effects, whereas others were characterized by sedating or soporific effects. Furthermore, the Greeks commonly used olive oil to absorb flavors from flower petals or herbs, as well as for cosmetic and medicinal purposes [5,6]. Pepper, cinnamon, and ginger, for example, were spices imported from the East by the ancient Greeks to the Mediterranean region. The Romans utilized spices as a food flavoring, cosmetic, and for therapeutic purposes. When the Roman Empire extended to new regions, they introduced pepper and other spices from the East to other civilizations [6]. Over time, their economic relevance stimulated the opening of trade routes, the emergence of civilizations in the East and West, and the great continental discoveries of the 15th and 16th centuries [7].

The flavors of spice plants are in the natural volatile oils of the plants, and usually the true taste is only developed after drying [8,9]. Spices are defined as products derived from plants and characterized by aromatic or pungent substances that are used to flavor foods. They add aroma and flavor to food and enhance the pleasure of eating. Generally, spices have a color that varies from black to shades of brown and red, with striking and pungent flavor [8,10]. Condiment species have their aromatic properties distributed in different organs of plants, such as in leaves (rosemary, marjoram, oregano, basil, mint, and parsley), flowers (clove, turmeric, lavender, and orange), fruits (peppers, star anise, and tamarind), roots (turmeric and ginger), twigs or bark of trees (cinnamon), bulbs (garlic and onion), seeds and grains (coriander, cumin, fennel, nutmeg, and sesame), and resin (myrrh). The oils from coconut, sesame, olive, and palm are also used for culinary purposes [8,10].

In traditional medicine, the spice plants are described for their antioxidant, antimicrobial, diuretic, antiseptic, anthelmintic, stimulant, anti-inflammatory, analgesic, and carminative properties [10,11,12]. Spice plants have been gradually used in developed countries as a source of bioactive substances [11]. Traditional communities seek herbs and spices for their potential health benefits and apply them as remedies and supportive agents in remedial practices. The Mediterranean diet is one of the richest in nutrient content, taste, and flavor in the world, and the spices used in this diet are described by its anti-diabetic, anti-inflammatory, anti-hyperlipidemic, and anti-hypertensive properties [12,13].

The bioactive compound content of spice plants varies depending on the harvest period, type of processing, drying, and storage, which also could considerably affect their therapeutic properties. For each species, there are specific conditions to obtain the appropriate composition of active compounds. Several spice plants have essential oils (Eos) described by their antioxidant activity, which may also vary depending on their chemical composition. In the present review, we exhibit a general overview of the main aspects related to the importance of commonly used spice plants that have been applied in traditional medicine around the world: *Cinnamomum zeylanicum* Blume (true cinnamon), *Mentha piperita* L. (peppermint), *Ocimum basilicum* L. (basil), *Origanum vulgare* L. (oregano), *Piper nigrum* L. (black pepper), *Rosmarinus officinalis* L. (rosemary), and *Thymus vulgaris* L. (thyme), and discuss the pharmaceutical, cosmetic, and food applications of bioactive compounds obtained from their EOs and their molecular mechanisms of action, focusing on their antioxidant activity.

## 2. Methods Applied in Determining Antioxidant Activity 

Essential oils (EOs) are formed by different organic compounds that contain conjugated carbon double bonds, as well as hydroxyl groups, which can donate hydrogen, thus inhibiting free radicals and minimizing oxidative stress [14,15]. Different in vitro chemical-based methods have been developed for determining the antioxidant activity of EOs and their natural or synthetic-derivative compounds. Among them, we can cite DPPH, ABTS, and hydroxyl assays, which are indicated to evaluate the radical scavenging activity of the organic compounds and have been usually applied to extracts, EOs, and isolated organic substances; *β*-carotene-linoleate model systems and thiobarbituric acid reactive substances (TBARS) assays, which are applied to evaluate foods and organic substances with lipid contents; as well as the ferric antioxidant power reduction (FRAP), cupric ion reducing antioxidant capacity (CUPRAC), and phosphomolybdenum (PM) assays, which are used to evaluate the reducing power of the analyzed compounds, EOs, or mixtures. The results of in vitro chemical-based assays are useful, low-cost, high-throughput, and have been widely applied to discover potential antioxidant sources. Nevertheless, to evaluate the antioxidant activity, it is recommended to use more than one assay [16]. Currently, there is a great variation in the methodologies described for antioxidant assays, such as DPPH and CUPRAC, showing different protocols for determining and interpreting the antioxidant activity of the analyzed sample. These methodological differences create biases in the comparison of the results. Additionally, the higher solubility of some standard compounds such as DPPH and *β*-carotene in organic medium (e.g., methanol and ethanol) are also limitations [16,17]. The TBARS assay is widely used to quantify lipid peroxidation, but presents some limitations such as the reaction of TBA with other compounds not related to lipid peroxidation [17]. The FRAP assay is usually indicated to biological samples with high phenolic content, but has limitations regarding the reaction time, which varies depending on the evaluated antioxidant compound [17]. However, biological tests, such as enzyme- [18] and cell-based assays [19,20] have been pointed out as interesting and confirmatory methods to validate the antioxidant activities in biological systems [20,21]. In this section, we present the main aspects related to the methods applied to determine the antioxidant activity treated in the scientific reports of this review. We also recommend that the readers consult some excellent reviews on in vivo and in vitro assays [17,20,22,23].

### 2.1. Chemical-Based Assays

#### 2.1.1. Radical Scavenging Assays

**DPPH radical scavenging assay:** DPPH (1,1-diphenyl-2-picrylhydrazyl) is a stable free radical which has been used to simulate the antioxidant activity of chemical constituents of EOs, extracts, and other substances from natural products [24]. The antioxidant compounds present in the EO react with the DPPH, thus converting it to 1-diphenyl-2-picryl hydrazine. Then, the antioxidant activity is measured in a spectrophotometer at 517 nm and calculated according to Equation (1), where A_i_ is the absorbance of the sample, and A_o_ is the absorbance of the control solution.

(1) $$\%\;\mathrm{Radical}\;\mathrm{scavenging}\;\mathrm{activity}=\frac{\left(A_{0}-A_{i}\right)}{A_{o}}\times 100$$

**ABTS radical scavenging assay:** ABTS (2,2′-azino-bis (3-ethylbenzothiazoline-6-sulfonic acid)) radical is widely used to assess antioxidant activity, especially in foods [25]. The ABTS radical scavenging assay initially consists of the conversion of ABTS (colorless) to ABTS^+^ (blue), which occurs by the addition of K_2_S_2_O_8_. In the presence of antioxidant compounds (phenolic compounds, thiols, and vitamins C and E) this cation is converted back to the neutral ABTS (colorless) form; therefore, the greater the disappearance of the blue color of the solution, the greater the antioxidant activity. Then, the antioxidant activity is measured in a spectrophotometer at 734 nm and calculated according to Equation (1). Finally, it can be compared to the activity of the Trolox (soluble vitamin E) and expressed in Trolox equivalent antioxidant capacity (TEAC). 

**Hydroxyl radical scavenging assay:** Hydroxyl radicals can be formed by the Fenton reaction, which occurs in the presence of transition metal ions in reduced form (e.g., Fe^2+^) and H_2_O_2_. The antioxidant activity of eliminating the hydroxyl radical is very important since this radical is very reactive towards sugars, amino acids, lipids, and even nucleotides. In this test, hydroxyl radicals are generated by ascorbate-hydrogen peroxide. Gallic acid is usually used as a control solution. After heating the mixture for 1 h at 37 °C, the absorbance is measured in an ultraviolet-visible (UV-Vis) spectrophotometer at 510 nm. Then, the hydroxyl radical scavenging activity is calculated according to Equation (1).

#### 2.1.2. Lipid Peroxidation Assays

**β-Carotene-linoleate model system:** The antioxidant activity is measured in an emulsified medium, through the co-oxidation of substrates [26]. This method consists of measuring the discoloration of an aqueous solution containing β-carotene and linoleic acid by reading the colored solution at 470 nm. This discoloration is due to the oxidation of β-carotene double bonds due to the action of radicals formed by the oxidation of linoleic acid. The butylhydroxytoluene (BHT) is usually used as a standard to determine the antioxidant activity. This method is usually applied to investigate the antioxidant activity of solutions or substances rich in lipids. However, the technique requires the use of high temperatures, so it cannot be applied in food or thermosensitive substances [27].

**Thiobarbituric acid reactive substances (TBARS) assay**: This method is performed to measure malondialdehyde (MDA) present in samples and MDA generated from the lipid peroxidation. In this test, a biological sample containing lipids is heated to 50 °C with 2-thiobarbituric acid, trichloroacetic acid, and HCl. Afterward, the MDA forms an adduct with two molecules of thiobarbituric acid, producing a pink colored complex, and its absorbance can be measured between 532 and 535 nm. The absorbance is proportional to the concentration of MDA generated. However, MDA is not the only product formed by lipid oxidation [28,29].

#### 2.1.3. Reduction Power Assays

**Ferric antioxidant power reduction (FRAP) assay**: The mechanism of this method is based on electron transfer. When the Fe^3+^ ion is reduced in an acid medium (pH = 3.6) to Fe^2+^ in the presence of 2,4,6-tripyridyl-s-triazine (TPTZ), the formation of a Fe^2+^-TPTZ complex (blue color) occurs, which absorbs in the region 593–595 nm. The acidic pH prevents the formation of hydroxides and oxides, keeping the iron soluble. It also increases the redox potential, which favors the reaction. The reducing capacity is related to the degree of hydroxylation and the degree of conjugation of the bonds present in the phenolic compounds found in extracts and EOs [30]. For analyses with volumes between 50 and 200 μL, a volume of FRAP reagent 0.2 to 5.0 mL is in general applied to perform the antioxidant activity assay. Trolox or gallic acid is used in the calibration curve and the blank is standardized with deionized water [31].

**Cupric ion reducing antioxidant capacity (CUPRAC) assay:** The CUPRAC assay is a quick and simple test applied to assess the antioxidant activity of foods, dietary polyphenols, and biological fluids. The bis(neocuproine)copper(II) chloride (Cu(II)-Nc) is the chromogenic oxidizing reagent used in this essay, which acts as an electron transfer agent. Antioxidant reagents (polyphenols, thiols, and vitamins C and E) present in food reduce Cu(II)-Nc to Cu(I)-Nc. The absorbance of the Cu(I)-Nc complex (orange-yellow) is measured at 450 nm. It is worth mentioning that the pH must be adjusted to 7 and the absorbance must be measured within 30 min [32].

**Phosphomolybdenum (PM) assay:** The PM assay consists of reducing Mo(VI) to Mo(V) (green complex) under heating and acidic pH conditions with the presence of the evaluated compounds of the sample. This methodology is used to determine the total antioxidant activity of plant extracts and EO. In general, ascorbic acid and rutin are used as references. The absorbance of the samples is read at 695 nm and the antioxidant activity is compared with a reference compound (e.g., vitamin C) [33].

### 2.2. Cell- and Enzyme-Based Assays 

Cell- and enzyme-based assays are considered biological tests indicated to confirm the initial screening performed with the in vitro chemical-based assays [34,35]. The cell-based antioxidant assays require cell line systems. Compared with the chemical-based assays, these methods are time-consuming, expensive, and more complex, but allow us to evaluate some aspects related to the pharmacokinetics, such as membrane uptake and metabolism, as well as the in vivo effectiveness of the analyzed compounds [17,20].

**Cellular antioxidant activity (CAA) assay**: These methods use cell lines, such as Caco-2, HepG2, IPEC-J2, and MCF-7 cells, and a biosensor to evaluate the antioxidant activity [36,37,38]. Some studies apply dihydrodichlorofluorescein (DCFH_2_, fluorescence probe dye) as a redox sensor, which oxidizes to fluorescent dichlorofluorescein (DCF) with the presence of peroxyl (ROO^•^) resulting from the decomposition of 2,2-azobis(2-methylpropionamidine) hydrochloride (AAPH). The method measures the ability of the analyzed compounds to inhibit the oxidation of intracellular DCFH_2_ that can be analyzed by fluorescence. The antioxidant activity is expressed in moles of quercetin equivalents. The emitted fluorescence is recorded every 5 min for 1 h with the absorbances λ_exc_ = 485 nm, λ_em_ = 535 nm [17,20].

**Inhibition of antioxidant enzymes:** These assays evaluate the antioxidant activity of compounds by analyzing the activity of enzymes that belong to the antioxidant defense system, such as catalase (CAT), superoxide dismutase (SOD), glutathione peroxidase, glutathione-S-transferase, and glutathione reductase (GR) [23,39]. Regarding GR, it regulates the balance between the oxidized (GSSG) and reduced (GSH) glutathione in the cell. The GSH inhibits the ROS and RNS, thereby contributing to the control of redox homeostasis. Based on that, the GSH/GSSG ratio is used as an index to evaluate oxidative stress [40].

## 3. Bioactive Compounds from EOs of Spice Plants

EOs are derived from plants and contain a high diversity of volatile, aromatic, and low-molecular-weight compounds that have been widely investigated for cosmetic and pharmaceutical purposes [41,42,43], and for use in the food industry as flavoring and preservatives [44]. EOs are obtained from all parts of the plant and they are described as liquid, volatile, limpid, and sometimes colored [45].

**Figure 1:**
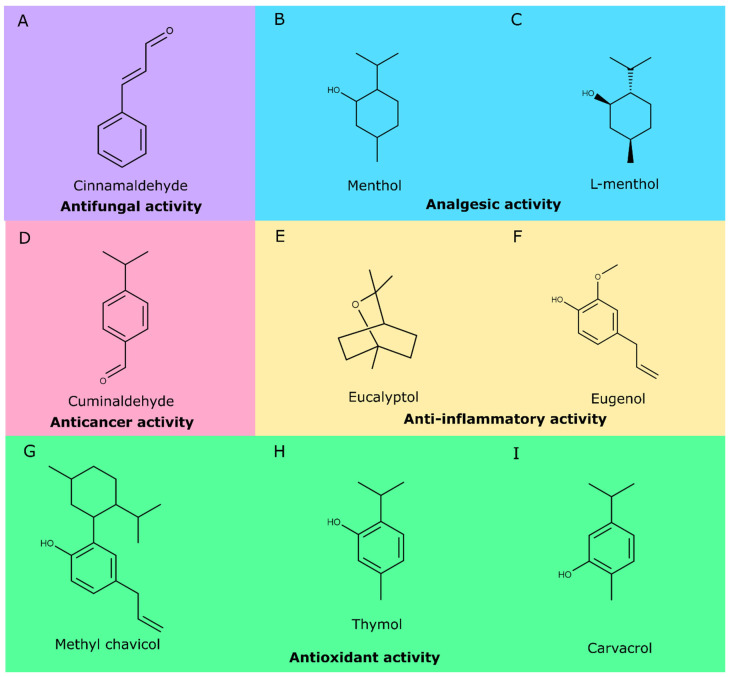
Natural compounds from essential oils and their reported biological activities with potential pharmaceutical applications.

The compounds from EO described with antioxidant activity represent a relevant fraction of their chemical composition [46]. These components have been indicated as an interesting source for the development and discovery of new bioactive compounds with pharmaceutical and cosmetic application due to their anti-inflammatory [47], antibacterial [48], antifungal [49,50], repellent [51,52], antioxidant [53,54,55], and neuroprotective [56] properties, as well as cytotoxic activities against cancer cell lines [57,58]. In addition, EOs and their constituents have been investigated as an alternative additive in the food industry [44,59] in contrast to synthetic antioxidants, such as butylated hydroxytoluene, butylated hydroxyanisole, and propyl gallate, that have exhibited undesired effects to human health [60,61]. Natural compounds from plant EOs which have shown biological activities with potential pharmaceutical applications include (*E*)-cinnamaldehyde (antifungal activity, Figure 1 panel A) [62]; menthol and L-menthol (analgesic activity, panels B and C) [63]; cuminaldehyde (anticancer activity, panel D) [64]; eucalyptol and eugenol (anti-inflammatory activity, panels E and F) [65,66,67]; and thymol, carvacrol, and methyl chavicol (antioxidant activities, panels G, H, and I) [68,69]. It is important to note that some well-known compounds found in EOs have been recently described with new biological activities, e.g., monoterpenes such as thymol, carvacrol, and *p*-cymene have been reported to reduce lung emphysema and inflammation [70] and eugenol has shown activity against several parasites with clinical relevance [71,72].

Regarding the antioxidant activities from these natural compounds, these are related to the ability to inhibit oxidative stress. Oxidative stress is disturbance in the oxidant-antioxidant balance that leads to the increase and consequent attack of reactive oxygen species (ROS), such as alkoxyl (RO**^•^**), superoxide anion (O_2_**^•^**), hydroxyl (HO**^•^**), and peroxyl (RO_2_**^•^**) radicals against the structural components of the cell and some reactive nitrogen species (RNS), such as nitric oxide (NO^•^) and peroxynitrite (ONOO^−^) [73,74]. The presence of oxidative stress is related to the development of several human disorders, such as Alzheimer’s disease and cancer [73].

Plants used as condiments have been reported as belonging to different botanical families and were described with a huge diversity of antioxidant compounds able to prevent oxidative stress [75]. These antioxidant compounds include geraniol, thymol, *p*-cymene, menthol, eucalyptol, and carvacrol. The antioxidant activity of some compounds has been pointed out due to the presence of hydroxyl groups in their structures [15]. In the next sections, we exhibit a general overview of the main aspects related to the importance of commonly used spice plants that have been applied in traditional medicine around the world: *C. zeylanicum* (true cinnamon), *M. piperita* (peppermint), *O. basilicum* (basil), *O. vulgare* (oregano), *P. nigrum* (black pepper), *R. officinalis* (rosemary), and *T. vulgaris* (thyme), and discuss the potential pharmaceutical, cosmetic, and food applications of bioactive compounds obtained from their EOs and their molecular mechanisms of action, focusing on their antioxidant activity.

### 3.1. Cinnamomum Zeylanicum (True Cinnamon)

**General information:** The true cinnamon (*C. zeylanicum*, *C. verum* Lauraceae), also known as Ceylon cinnamon, is a plant originating from India and Sri Lanka, where it is used as a spice and in traditional medicine to treat different human diseases [76]. Cinnamon spice is obtained from different parts of the plants, such as the trunk bark, root, leaves, and flowers [77].

**Traditional uses and applications:** The plant is used in traditional medicine in different world regions to treat various digestive, respiratory, and gynecological diseases [78]. Parts of cinnamon are widely used in culinary applications as a condiment and flavoring in the preparation of foods, such as liqueurs and chocolates [76]. Due to the antimicrobial activity, the total content of its EO has been also investigated in the vapor phase for heritage textile disinfection [79] and as a wine preservative [80].

**Chemical composition:** The main chemical constituents found in true cinnamon EO, vary according to the plant organ, geographical origin, environmental conditions, extraction, and drying methods, but in general, it contains, as major compounds, (*E*)-cinnamaldehyde and (*E*)-cinnamyl acetate (Table 1). Some compounds such as cinnamaldehyde and camphor have been reported to be the major components of volatile oils from stem bark and root, respectively. In contrast, eugenol has been reported as a major component of the leaves [81].

**Bioactivity and bioactive compounds:** The true cinnamon EOs have been reported by their anti-fungal, antibacterial, antioxidant, as well as cytotoxic properties against cancer cell lines [82,83,84]. Recently, a study investigated the antimicrobial activity of *C. zeylanicum* EO ((*E*)-cinnamaldehyde: 45.62%) and its main components (cinnamaldehyde and eugenol) and demonstrated that the EO showed significant inhibitory activity against bacteria and fungi strains with MIC values ranging from 20 to 120 µg∙mL^−1^, depending upon the species. Cinnamaldehyde and eugenol were identified as the most active antibacterial compounds [85]. Similarly, studies have also demonstrated that cinnamaldehyde is active against *Mycobacterium tuberculosis*, contributing to disturbances in bacteria detoxification mechanisms, redox homeostasis [86], as well as cell membrane stress [87].

Regarding the anticancer activity, a study demonstrated that cuminaldehyde, a component from the true cinnamon tree’s bark, has cytotoxicity against human colorectal adenocarcinoma cells (COLO 205) through targeting Topoisomerase I and II, which are relevant molecular targets for cancer therapy [64]. Unlu et al. [88] demonstrated the cytotoxic activity of EOs of *C. zeylanicum* composed of major compounds (*E*)-cinnamaldehyde (68.95%), benzaldehyde (9.94%), and (*E*)-cinnamyl acetate (7.44%) against F2408 (normal rat fibroblasts) and 5RP7 (H-ras active-rat fibroblasts) cell lines.

A study identified that eugenol has anti-proliferative and anti-metastatic activity against triple-negative and HER2 positive breast cancer cells; in both cases the authors identified that the compound increased the expression of genes involved with apoptosis, such as *caspase3*, *caspase7*, and *caspase9* [89]. Interestingly, a study showed that the combined treatment of eugenol and 5-fluorouracil displayed a cytotoxic effect against HeLa cell lines, inducing apoptosis [90].

Recently, Tepe and Ozaslan [91] investigated the anti-Alzheimer’s, anti-diabetic, and skin whitening effects of *C. zeylanicum* (Blume) EO and its major components (*E*)-cinnamaldehyde (81.39%) and (*E*)-cinnamyl acetate (4.20%). The EO and the major compounds, (*E*)-cinnamaldehyde and (*E*)-cinnamyl acetate, showed over 78.0% anticholinesterase activity. In the inhibition assay of monoamine oxidase A and B (related to Alzheimer’s disease), the EO showed 96.44% and 95.96%, respectively, while (*E*)-cinnamaldehyde displayed 96.32% and 96.29%, respectively. The inhibitory activities of the *C. zeylanicum* EO, (*E*)-cinnamaldehyde, and (*E*)-cinnamyl acetate were found to be in the range of 7.29–21.30% on α-amylase (diabetic). It is also interesting to note that the isolated compounds (*E*)-cinnamaldehyde (83.75%) and (*E*)-cinnamyl acetate (45.58%) showed higher activity in the tyrosinase inhibition assay when compared with the EO (18.08%).

Concerning the methyl cinnamate, a study investigated the molecular mechanism of the inhibition of CCI-induced mechanical and thermal hypersensitivity involved with neuropathic pain. The authors identified that methyl cinnamate reduces pain hypersensitive behaviors and CCI-induced upregulation of spinal AMPA receptors through activation of AMP-activated protein kinase [92].

**Antioxidant activity:** Lin et al. [85] evaluated the antioxidant potential of 45 EOs of different species against the DPPH radical. In this study, they identified that EOs of cinnamon and clove bud showed the highest inhibition rates (approximately 96%), and these oils showed more than 80% of eugenol in their composition. Some recent studies have described the antioxidant activity of the true cinnamon EO (Table 1). The EO also exhibited remarkable antioxidant activity when assessed by DPPH radical scavenging and β-carotene bleaching assays, compared to BHA, α-tocopherol, and BHT. 

In vitro studies have demonstrated that eugenol possesses satisfactory antioxidant and radical-scavenging activities [93]. Interestingly, the antioxidant activity of *C. zeylanicum* (Blume) EO was investigated using PM and CUPRAC assays, and the EO showed higher activity than the main components (*E*)-cinnamaldehyde (81.39%) and (*E*)-cinnamyl acetate (4.20%), whereas in the FRAP assay the oil exhibited higher activity than (*E*)-cinnamyl acetate [91].

Kallel et al. [83] investigated the cytotoxic activity of *C. zeylanicum* EO with >77% of polyphenols using an MTT assay against HeLa and Raji cell lines. They found that the EO inhibits the proliferation of both cell lines presenting IC_50_ values of 0.13 and 0.57 μg∙mL^−1^, respectively. The authors also investigated its potential as an antioxidant using PM, DPPH, and hydrogen peroxide radical scavenging assays, and compared it with synthetic BHT and ascorbic acid as positive controls. The potency of the PM assay was of the order of 108.75 ± 32.63 mg of essential oil/equivalent to 1 mg of ascorbic acid in terms of antioxidant power, and the antioxidant activity of DPPH and hydrogen peroxide radical scavenging assays were 21.3% and 55.2%, respectively.

### 3.2. Mentha Piperita (Peppermint)

**General information:** Peppermint (*M. piperita,* Lamiaceae) is also known as *Mentha balsamea* Wild. The plant is native to Europe and the Middle East, but it is cultivated in different parts of the world [94].

**Traditional uses and applications:** Leaves of peppermint are applied as a condiment in some food dishes and as flavoring for ice creams and liqueurs. In traditional medicine, EOs and extracts of *Mentha* L. have been widely used to treat inflammation of the oral mucosa, cold, musculoskeletal pain, gastrointestinal and respiratory diseases [95]. Those uses have been related to the presence of secondary metabolites in its EOs, such as monoterpenoids and phenolic compounds [96].

**Chemical composition.** The chemical composition of peppermint EO varies according to the extraction method, environmental conditions, and geographical origin, but, in general, it contains as the major constituents: menthol, epoxyocimene, linalool, menthone, eucalyptol, and *neo*-menthol (see Table 2).

**Bioactivity and bioactive compounds:** The EOs of peppermint have been reported to have antioxidant [97], anti-inflammatory [57], antibacterial [53], antifungal [98] effects, as well as cytotoxic activity against human cancer cell lines [57]. *M. piperita* EO has been investigated in the treatment of edema, bacterial infection, and inflammation in animal models [99].

Menthol is one of the most bioactive compounds and has remarkable biological activities including analgesic, anti-inflammatory, and antimicrobial [100]. Menthol, when in moderate concentrations, inhibits the irritancy caused by capsaicin [101] and its high concentrations induce analgesia [102]. Zaia et al. [103] investigated the anti-inflammatory activity of menthol and menthone in *Schistosoma mansoni* infection and identified that the blood levels of pro-inflammatory cytokines, such as IL-4 and IL-10 were reduced in animal models, thus contributing to mitigate the physio-pathological effects of parasite infection.

Several studies have reported the antimicrobial activity of menthol and its derivatives against bacterial species [104,105]. Additionally, L-menthal and L-menthol have been reported with bioactivity against bacterial biofilms [106]. The perturbation of the permeability of the lipid fraction of the plasma membrane has been reported as a possible mechanism of antibacterial action of some EO compounds, such as (*D*)-menthol [107].

Benzaid et al. [53] investigated the antimicrobial activity of *M. piperita* L. EO against bacterial strains and *Candida albicans* yeast. The EO showed, as major compounds, menthol (32.93%), menthone (24.41%), cis-caran (8.08%), and eucalyptol (1,8-cineole, 7.89%) and was shown to reduce the growth and biofilm formation of different bacteria species and *Candida albicans* yeast. The percentage of growth reduction was ranged from 40% to almost 100%, depending on the tested microorganism.

**Antioxidant Activity:** Recently, studies have shown the antioxidant activity of peppermint EO (Table 2). Singh et al. [108] evaluated the antioxidant DPPH free radical scavenging activity of EO and different extracts of *M. piperita*. The inhibition percentages varied between 70% and 93%, with the highest values obtained for EO (92.6% ± 6.8%) and chloroform extract (91.8% ± 5.8%) and the lowest value for aqueous extract (70.3% ± 6.1%). Wu et al. [37] evaluated the chemical composition and antioxidant activity of EOs from three mint species: peppermint (*M. piperita*), native mint (*M. spicata*), and Scottish mint (*M. gracilis*). All analyzed EOs showed Fe^3+^ reducing activity, and all of them showed a reduction activity with the increase in the concentration of EO (between 0 and 200 mg∙mL^−1^). The average maximum effective concentration (EC_50_) of mint species found were the following: 22.7; 22.9, and 23.4 mg∙mL^−1^, for peppermint, Scottish mint, and native mint, respectively. The authors also evaluated the antioxidant activity of these three species EOs in vivo using CAA with the IPEC-J2 cell line and they found that the maximal cellular antioxidant activity for peppermint EO was 5 μg·mL^−1^ and for native mint and Scotch spearmint EOs was 100 μg·mL^−1^. The main chemical constituents of EOs varied for each species, which may be responsible for the variation in their activities. In this study, the peppermint EO presented the main compounds: menthol (38%), L-menthol (22%), eucalyptol (1,8-cineole) (6%), and neo-menthol (4%).

It is important to highlight that some studies have reported that the radical scavenging activity of peppermint EOs is associated with the presence of menthol and menthone, which contains the hydroxyl radical (-OH) [37,57]. However, Ramos et al. [109] evaluated the antioxidant activity of peppermint EO containing linalool as a major constituent (51.80%) and the oil possessed a significant radical scavenging activity in the concentration of 100 mg·mL^−1^ with % AA 79.9 ± 1.6.

### 3.3. Ocimum Basilicum (Basil)

**General information**: *O. basilicum* (Lamiaceae), commonly known as basil or sweet basil, is an aromatic plant native from the Asian region, but it has been introduced worldwide [110]. Basil is cultivated in East Asia, Europe, America, and Australia, mainly for EO production. Basil cannot be stored for long periods after harvest, as this will result in a reduction in quality. When basil is cultivated for EO extraction, the plant is harvested during full bloom and the harvest time is fundamental to obtain appropriated quality and satisfactory yields [110,111]. Basil is the most important among all *Ocimum* spp. and its EO has food applications [112].

**Traditional uses and applications:** Basil has been used in traditional medicine for the treatment of headache, cough, and cold. Its tea has been indicated for the treatment of dysentery, nausea, flatulence, and also as anti-convulsant, anti-hyperlipidemic, and anti-inflammatory. *O. basilicum* is also reported by its uses as a flavoring agent in foods, such as soups, cheeses, vinegar, oils, food preservation, aromatherapy, and perfumery industries [110,111,113,114]. Basil EO can be used in combination with other spices to prepare condiments, bakery products, ice creams, and others. The components of basil EO were recognized as safe by the US FDA and European Commission [112].

**Chemical composition:** The chemical composition of basil EOs can vary according to the cultivars, geographical distribution, growth stage, harvesting season, cultivation conditions, and other factors. In general, the basil oils have been classified in the function of their major compounds: linalool, methyl chavicol (estragole), methyl cinnamate, and eugenol (Table 3) [110,111,112,115].

**Bioactivity and bioactive compounds:***Ocimum* species have shown antihyperglycemic potentials in vitro and in vivo [113], and anticancer activities [116]. *O. basilicum* EO has been described by its antimicrobial [117,118], larvicidal [119], and antioxidant activities [28], as well as, cytotoxicity against cancer cell lines [120].

Regarding the application of *O. basilicum* EO, it has been described by its larvicidal activity against mosquitos with medical relevance, such as *Aedes albopictus* and *Anopheles subpictus* [121]. Interestingly, linalool and methyl cinnamate have been reported with repellent, larvicidal, and insecticidal activities [122]. Methyl chavicol, the major compound identified in this oil, has been described by its antimicrobial and antioxidant activities [28]. Recently, a study identified that this compound and its analogs inhibit the human pancreatic lipase, thus showing a potential application in the treatment of lipid disorders [68].

Regarding microbial activity of *O. basilicum* compounds, it is well-known that eugenol exhibits antifungal activity against different fungal species, such as *Candida albicans* and *Cryptococcus neoformans* [123] and it has been suggested that this compound inhibits the ergosterol biosynthesis, thus interfering with the integrity of the cell membrane [124]. Eugenol has also shown activity against protozoa with clinical relevance, such as *Giardia lamblia*, *Schistosoma mansoni*, and *Haemonchus contortus* [71,72].

**Antioxidant activity**: Table 3 reports studies describing the antioxidant activity and chemical variation in the EOs of *O. basilicum* specimens. Ahmed et al. [115] studied specimens of *O. basilicum* from three different locations in Egypt (Assiut, Minia, and Beni Suef). The percentage of the major components varied according to the geographical origin. EOs obtained from Assiut were characterized by linalool (31.66%), methyl chavicol (17.37%), and methyl cinnamate (15.14%). The basil EO from Minia showed high levels of linalool (28.18%), and methyl chavicol (16.98%). Linalool (27.64%), methyl chavicol (5.96%), and methyl cinnamate (10.49) were the compounds with a percentage greater than 10% in the EO composition obtained from Beni Suef. The EOs were subjected to the DPPH assay and its radical scavenging activity varied between IC_50_ = 11.23 mg∙mL^−1^ to 55.15 mg∙mL^−1^. Those values were lower than the positive control, BHT (IC_50_ = 6.80 mg∙mL^−1^). The highest antioxidant activity was observed for the EO extracted from plants collected in Minia and this was probably due to an increase of eugenol content (7.34%), approximately two or three times greater than in the other samples.

Some varieties of *Ocimum* spp. were compared on the basis of their chemical composition. Linalool was the major compound for all varieties, with contents from 51.1% to 59%. Methyl chavicol was the predominant constituent in the EOs of Italian large leaf, purple ruffles, and green purple ruffles (46.4–59.5%). Holy basil and cinnamon basil were characterized by a high percentage of methyl eugenol (74.7%) and E-methyl cinnamate (45.9%), respectively. ABTS and FRAP assays were conducted to evaluate the antioxidant activities of the EOs, and these activities were associated with the linalool-eugenol chemotypes. Osmin purple EO showed the highest radical scavenging activity (996.7 ± 137.1 μmol Trolox∙mL^−1^ EO-ABTS; 1262.9 ± 8.0 μmol AA∙mL^−1^ EO-FRAP), which was associated with the highest levels of eugenol (14.7%). When ABTS and FRAP assays were conducted, the antioxidant activity of methyl eugenol was higher than that of methyl chavicol, which could be an effect of the additional methoxy group. Eugenol is an important EO component due to its high antibacterial and antioxidant activities [125].

Different authors have employed the DPPH assay to evaluate the antioxidant activities of basil EO. Sundararajan et al. [119] encapsulated the basil EO in a Polysorbate 80 matrix and displayed its antibacterial, antioxidant, and larvicidal activities against *Culex quinquefasciatus*. From the DPPH scavenging activity assay, the EO showed an IC_50_ = 13.21 μg∙mL^−1^, while nanoemulsion and gallic acid showed IC_50_ = 10.47 μg∙mL^−1^ and 7.90 μg∙mL^−1^, respectively, indicating that the EOs exhibited good antioxidant activity. Baj et al. [126] designed a mixture of marjoram (16% of linalyl acetate, 14.7% of linalool), basil (75.1% of methyl chavicol) and rosemary (16.6% of α-pinene, 15.2% eucalyptol, and 15.1% of camphor) EOs and evaluated the antioxidant activity. They observed an intermediate activity to the pure basil EO (48.6%). When evaluating the mixture, the highest value for antioxidant activity (83.9%) was achieved in the combination 2/3 of marjoram, 1/3 of rosemary, and 0 of basil EO. Stanojevic et al. [127] described the chemical composition, antioxidant, and antimicrobial activity of basil EO which was characterized by linalool (31.6%) and methyl chavicol (23.8%). The DPPH scavenging activity assay indicated that basil EO may be an alternative antioxidant, with applications in food and pharmaceutical industries. The highest antioxidant activity was reached after 90 min of incubation (EC_50_ 2.38 ± 0.10 mg∙mL^−1^) while BHT was 0.021 mg∙mL^−1^.

Shiwakoti et al. [128] conducted a study to evaluate different extraction methodologies (hydrodistillation and steam distillation) on the yield, antioxidant activity, and chemical composition of two *Ocimum* spp. Basil EO was characterized by high contents of linalool (23.32–28.10%), methyl chavicol (15.74–20.64%), and methyl cinnamate (19.31–20.20%). Changes in distillation methodology did not affect the antioxidant activity, which varied from 1735 ± 281 to 1878 ± 247 µM TE/g. 

Oxygen introduction during the extraction of basil, lemongrass, and lemon was conducted and the ORAC method was employed to evaluate the antioxidant activity of the EOs obtained. The results showed that the addition of oxygen during the hydrodistillation promoted an increase in the antioxidant activity, which was related to the increase in oxygenated terpenes [129]. 

### 3.4. Origanum Vulgare (Oregano)

**General information**: Oregano refers to a variety of different plant species that belong to six different botanical families, that share a similar odor and flavor, and which are used as condiments around the world. The Lamiaceae and Verbenaceae are pointed out as the most economically relevant families, and the *O. vulgare* (Lamiaceae) belongs to the most studied species of oregano [131]. *O. vulgare* is a native plant from the Mediterranean region, but it is cultivated in different parts of the world.

**Traditional uses and applications**: The dried leaves and flowers of *O. vulgare* are widely used as condiments around the world to flavor different kinds of foods, such as pizzas, salads, sausages, fried potatoes, and have also been employed as an ingredient of typical Mediterranean, Mexican, and Italian cookery. *Origanum* species have also been used to treat different human diseases, such as wounds, cough, and skin and gastrointestinal problems [132]. Due to the different applications of oregano flowers and leaves as a condiment, its EOs have been intensively investigated as a food preservative [133,134,135]. Additionally, different studies have demonstrated the antimicrobial activities of oregano EOs against bacterial strains with medical relevance [136,137,138]. Despite the antioxidant activities, recent studies have demonstrated their antidiabetic, anti-inflammatory, and cytotoxic activities against cancer cell lines [47,131,139,140].

**Chemical composition:** The volatile compounds of oregano EOs vary greatly, and are influenced by drying methods [141] and origin species [142]. Regarding *O. vulgare* EO in general, it has exhibited as major constituents some phenolic compounds, such as *p*-cymene, carvacrol, linalool, and thymol, which have been related to the antioxidant activity (Table 4) [131,143,144].

**Bioactivity and bioactive compounds**: *p*-cymene, a natural antioxidant, has been pointed out as a neuroprotective agent [145]; and carvacrol and thymol have been widely investigated by the food industry as additives due to their antioxidant activities [146]. Carvacrol acetate and thymol obtained from oregano species have also been reported due to their decreasing activity of ROS levels produced by macrophage cells stimulated with lipopolysaccharide [147]. Oregano EO and its major compounds, such as carvacrol, thymol, and *p*-cymene have been reported with acaricidal activity against *Psoroptes cuniculi* [148].

Regarding the antimicrobial activities, the EO of different oregano species has shown inhibitory activities against bacterial and fungal strains, which indicates their potential application in the food industry as a preservative [149,150]. The antimicrobial activity has been pointed out due to the synergic effect of constituents of oregano EO and fractions, especially when carvacrol and thymol are present [140].

**Antioxidant activity:**Table 4 exhibits some recent studies about the antioxidant activity of *O. vulgare* EOs. The antioxidant activity of the EO from two *O. vulgare* subspecies (subsp. *vulgare* and subsp. *hirtum*) was evaluated using different methods, such as free radical scavenging (DPPH and ABTS assays) and reducing power (FRAP and CUPRAC assays). They identified that the radical scavenging capacity and the reducing power of *O. vulgare vulgare* (thymol: 58.31%) showed significantly higher activities (*p* < 0.05) than *O. vulgare hirtum* (linalool: 96.31%). The authors also argue that thymol and carvacrol are excellent reductants [151].

Drying methods greatly influence the yield and antioxidant activity of oregano EO. Recently a study demonstrated that the highest oil yield and the highest antioxidant activity values were obtained from shade dried *Origanum* species. The lowest EO yield and the lowest antioxidant activity for *O. vulgare* were found in fresh plants. *O. vulgare* EO, with about 45% carvacrol, showed the highest DPPH anti-radical activity when obtained by shade drying (31.48%) followed by oven drying (26.19%) [152]. Studies have also demonstrated, using different methods such as DPPH and FRAP assays, that the antioxidant activity of *O. vulgare* EO varies according to the geographical region [143,153].

### 3.5. Piper Nigrum (Black Pepper)

**General information**: *P. nigrum* (black pepper, Piperaceae) is one of the world’s most widely used spices and it is distributed throughout the tropics and subtropics, as in Southwestern India. In Brazil, this plant has confirmed occurrences in different phytogeographic domains, such as the Amazon, Atlantic Forest, and Pantanal [156,157].

**Traditional uses and applications**: *P. nigrum* has been used for centuries to treat different human diseases, such as migraine, intermittent fever, gastrointestinal problems, and muscular pain. Its fruits are popularly used as a hot and pungent spice for flavoring food. Its fruits are usually dried and used as spices and seasonings [158,159]. Black pepper is also used to produce pepper oil and oleoresin, for the production of foods and also for perfumery [156,157,160]. Vitamins A, C, E, K, choline, folic acid, pyridoxine, riboflavin, thiamin, and niacin can be found in the black pepper constitution. *P. nigrum* also has some minerals, such as copper, calcium, magnesium, manganese, iron, phosphorus, and zinc [75].

**Chemical composition:**Table 5 exhibits different compositions and the investigated properties related to the *P. nigrum* EO. The (*E*)-caryophyllene is the major compound found in the EOs of *P. nigrum*, and β-thujene and limonene are also described in their chemical composition, but in smaller proportions.

**Bioactivity and bioactive compounds**: The literature shows an extensive list of studies involving the fruits of *P. nigrum*, but most of them describe biological tests of black pepper extracts [75,161,162].

(*E*)-caryophyllene (sesquiterpene) is the major chemical constituent of black pepper EO, but it is also found in several plants. Recently, a study has reported its potential application for the treatment of Alzheimer’s disease. The authors demonstrated that this compound inhibited amyloid β (Aβ) oligomer-induced neuroinflammation in BV-2 microglial cells by the inhibition of prostaglandin E2 production and nitric oxide, and concomitantly increased the expression of inducible nitric oxide synthase and cyclooxygenase-2, as well as significantly decreasing the secretion of pro-inflammatory cytokines such as IL-β, TNF-α, and IL-6 [163]. Another study demonstrated that (*E*)-caryophyllene prevents osteoblast dysfunction, the main cause of age-related bone loss. The authors found that it increased collagen content, alkaline phosphatase activity, osteocalcin production, and mineralization, which are involved in the stages of osteoblastic differentiation, thus promoting the formation of a mineralized extracellular matrix [164].

Regarding anticancer activity, different studies have reported that D-limonene induced autophagy and apoptosis in cancer cell lines [165] and enhanced the expression of caspase-3 involved with apoptosis [166]. Similar studies demonstrated that limonene has demonstrated cytostatic activity against cancer cell lines through cell cycle arrest [167].

Regarding bioactivity against parasites with medical relevance, a study has demonstrated that α-terpinene, one major constituent of *P. nigrum* EO, possesses a trypanocidal activity against *Trypanosoma evansi* in infected mice, and when associated with the standard drug, diminazene aceturate, the natural compound showed a satisfactory curative efficacy [168].

**Antioxidant activity**: Different methodologies have been used to evaluate the antioxidant activity of *P. nigrum* EOs (Table 5). Black and white peppers were harvested from five different provinces of China and the antioxidant activity was evaluated. It was observed that the DPPH radical activity of black and white pepper EOs was significantly different. According to the authors, the radical scavenging activity of *P. nigrum* EOs indicates its strong protective role against oxidative diseases and indicated its use as a natural antioxidant or food supplementation. For antioxidant activity assays, black pepper EOs were slightly superior to white pepper [160].

Rakmai et al. [169] subjected the encapsulated black pepper EO in hydroxypropyl-β-cyclodextrin (HPβCD) to physical-chemical characterizations, and evaluated the antioxidant and antibacterial activities. For free and encapsulated black pepper EO samples, the DPPH scavenging capacity increased with increasing sample concentrations (5–50 mg∙mL^−1^). Black pepper oil showed 54% (50 mg∙mL^−1^) inhibition using DPPH assay. The inclusion complexes provided a lower antioxidant activity when compared to the activity of the free black pepper EO and this behavior was associated with the block of the groups of active compounds during reaction with DPPH radicals, promoted by HPβCD.

*P. nigrum* EO was submitted to determination of the inhibition of lipid peroxidation, FRAP assay, superoxide radical scavenging, DPPH radical scavenging, and hydroxyl radical scavenging activities assays. The major compound in the EO of black pepper was (*E*)-caryophyllene (23.98), limonene (14.36%), α-terpinene (13.26%), γ-terpinene (13.26%), and caryophyllene oxide (8.68%). Concerning antioxidant activities, the results revealed that black pepper EO presents potential health benefits by scavenging the free radicals and minimizing inflammation and pains formed due to tissue injury. Oral administration of black pepper EO increased superoxide dismutase, glutathione, and glutathione reductase enzyme levels in the blood of mice. The study provided information about the potential therapeutic applications o black pepper EO [170].

Andriana et al. [157] and Bagheri et al. [171] evaluated the antioxidant activity of *P. nigrum* EO, rich in (*E*)-caryophyllene. The former used DPPH and ABTS assays and reported IC_50_ = 1.15 ± 0.08 mg·mL^−1^ (DPPH) and 1.74 ± 0.03 mg·mL^−1^ (ABTS). According to them, the EO obtained presented lower inhibitory effects than the positive control, BHT. Bagheri et al. [171] reported the antioxidant activity, applying the DPPH assay and an optimization procedure to compare the EC_50_ of the EOs and extracts obtained by hydrodistillation and supercritical fluid extraction, respectively. They obtained EC_50_ = 316.27 ± 0.12 µg·mL^−1^ for the EO and EC_50_ = 103.28 ± 0.05 µg·mL^−1^ for the extract and explained that the better results of antioxidant activities obtained in the extract could be related to the degradation of some antioxidant compounds present in EOs due to the enzymatic activity in the wet plant material.

### 3.6. Rosmarinus Officinalis (Rosemary)

**General information:***R. officinalis* (Lamiaceae) is native to the Mediterranean region, but it is distributed in all parts of the world, mainly due to its medicinal, culinary, food preservative, and cosmetic applications [172,173,174,175].

**Traditional uses and applications:** Fresh and dried leaves of *R. officicnalis* are commonly used as a condiment for flavoring food, herbal tea, and as a food preservative. Rosemary extracts are employed as a natural antioxidant to improve the shelf life of foods [173,174]. In general, the top flowering aerial parts including leaves, twigs, and inflorescences are mainly used to obtain the EOs and extracts [176]. Rosemary has been used in culinary applications to enhance and modify flavors, and in traditional medicine worldwide to treat different human diseases. In traditional Chinese medicine, it was used for headaches, and the Greeks and Europeans used the rosemary extract and EO to strengthen the memory, as a tonic, stimulant, and in the treatment of nervous tension. In Brazil, rosemary was described as an abortive agent in traditional medicine [172,174]. *Rosmarinus* spp. have been used in traditional medicines as an antispasmodic, diuretic, antiepileptic, carminative, renal colic, antirheumatic, expectorant, for diabetes, dysmenorrhea, heart diseases, and for relieving respiratory disorders [177]. In the United States and Europe, rosemary is the only spice commercially available for use as an antioxidant. Products based on rosemary are marketed in an oil-soluble form, in dry powder, and in water-dispersible or water-miscible formulations [178]. Rosemary extract was approved as a safe natural antioxidant for food preservation in the European Union [174,179]. Rosemary is an effective food preservative and presents low toxicity levels. Also, this species is strongly described by the antioxidant, antibacterial, and antifungal activities associated with its extract [14].

**Chemical composition**: Concerning the chemical composition of *R. officinalis*, the EOs were characterized by high levels of eucalyptol and camphor. The chemical compositions of *R. officinalis* EO vary, but it is very common to find the constituents eucalyptol, β-pinene, α-pinene, borneol, γ-cadinene, α-terpineol, myrcene, and camphene (Table 6).

**Bioactivity and bioactive compounds:** EOs and extracts of *R. officinalis* are sources of natural compounds characterized by several biological activities [180]. The biological properties in rosemary are attributed to phenolic compounds. The content of these antioxidants in the leaves is a function of different variables, such as seasonality, environmental conditions, species, and growing origin [172,181,182,183]. Nieto et al. [184] highlighted that antioxidant compounds present in rosemary extracts and EOs delay lipid oxidation in biological systems and food. Nevertheless, they described that the antioxidant activity of rosemary depends on the fruiting stage, nature of the extracts, extraction method, presence of an inhibitor, synergistic effect, and the concentration of active extract components.

Eucalyptol (1,8-cineole) is the most frequent major constituent of *R. officinalis* EO, and it has well-established anti-inflammatory and antinociceptive activities [185,186]. Studies have demonstrated that its anti-inflammatory activity occurs by suppression of lipopolysaccharide-induced proinflammatory cytokine production through the action of TNF-α, IL-6, NF-κB, and IL-1β, and reduction of oxidative stress through radical scavenging activity and regulation of signaling pathways [187].

**Antioxidant activity:** Different studies have evaluated the antioxidant activity of rosemary EOs to find new applications or even potentiate its antioxidant effect (Table 6). Baj et al. [126] utilized the simplex-lattice mixture method for the design of EO mixtures with potent antioxidant properties. Kowalski et al. [5] analyzed the effect of aromatization of rapeseed oil with rosemary on the content of volatile substances and evaluated the antioxidant properties of the aromatized oils. They observed that the best method of aromatization was the direct addition of EO to rapeseed oil and that the oils aromatized using the maceration method were characterized by higher antioxidant activity. Mezza et al. [188] obtained rosemary EO fractions using molecular distillation and evaluated their antioxidant activity and the effect on oxidative stability of sunflower oil. They concluded that the addition of rosemary EO fractions to sunflower oil improved the stability of the food product, avoiding lipid oxidation. Several studies demonstrated the antioxidant, antibacterial, and antileishmanial activities of rosemary EO [48,189,190]. Pistelli et al. [178] evaluated the antioxidant activity of EOs obtained from five different *R. officinalis* cultivars: *R. officinalis* ‘Alba’; *R. officinalis* ‘Corsican Blue’; *R. officinalis* ‘Israeli’; *R. officinalis* ‘Blue Rain’; *R. officinalis* ‘Majorca Pink’, and *Rosmarinus x lavandulaceus* Noë (a hybrid of rosemary) using FRAP and DPPH assays. The hybrid showed the highest antioxidant activity regardless of the method applied. Its EO was characterized by camphor (24.2% ± 0.5%), myrcene (15.3% ± 0.6%), and α-pinene (10.8% ± 0.4%). Selmi et al. [191] evaluated the protective effects of rosemary EOs on alloxan-induced diabetes and oxidative stress in rats, and also evaluated the antioxidant activity of kidney enzymes, such as SOD and CAT; and the DPPH radical scavenging activity. The authors found that the EOs significantly decreased enzyme concentrations and presented a high scavenging capacity. The analyzed *R. officinalis* EOs showed as major constituents 1,8-cineole, α-thujone (*E*)-caryophyllene, camphor, and α-pinene.

Wang et al. [192] described the variety in the chemical composition of the rosemary EO collected from China and Iran and their antioxidant activities. The samples grown in China presented better antioxidant activity and higher phenol content. Eucalyptol was the major compound for all samples of EO, followed by camphor, which presented values of 16.27% on average for China regions and 23.42% for the sample from Iran.

The results showed different levels of antioxidant activity, which was possibly associated with variations in the contents of chemical constituents. Several compounds present in EOs have a synergistic effect, which increases their antioxidant activity [193].

### 3.7. Thymus Vulgaris (Thyme)

**General information:** Thyme (*T. vulgaris*, Lamiaceae) is an aromatic and flowering plant, native to southern Europe and the Mediterranean region [194].

**Traditional uses and applications:** Thyme is widely used in cooking to flavor meat, salads, and soups. The leaves can be used as a decorative green herb in culinary art. *Thymus* species are rich in bioactive compounds and have been described for centuries as a spice, insecticide, herbal tea, and as incense due to their aromatic and medicinal properties. The EOs of *Thymus* spp. are usually used in traditional medicine due to the presence of their natural preservative compounds [195,196]. Dry and fresh leaves of thyme have been used in traditional medicine for the treatment of gastrointestinal, respiratory, and skin disorders. The species of *T. zygis*, *T. serpyllum*, and *T. pulegioides* are also used in different regions for similar purposes. Some commercial uses of these species are EOs products, oleoresins, landscape plants, and herbs [194]. Thyme EOs have been described as antimicrobial [197,198,199] and antioxidant [200,201]. Due to its antimicrobial and antioxidant properties, the thyme EO is an appropriate food additive and a flavor enhancer for foods and beverages, and is used for pharmaceutical, perfumery, and cosmetic purposes [194,195,196,199,202,203,204]. Thyme is a strong component in culinary and food processing due to its odor, taste, and antimicrobial activities. Those properties are important and depend on product requirements, processing parameters, and foodstuffs [194].

**Chemical composition:** The chemical composition of *Thymus* EO depends on the organ origin, environmental conditions, extraction methods, and other variables, but in general it has been described as containing, as its major compounds, monoterpenes, such as *m*- and *p*-cymene, and monoterpenoids, such as thymol and carvacrol [204,205]. Table 7 shows the majority of natural compounds, methodologies for determining the antioxidant activity, and some investigated applications of *T. vulgaris* EO. For all EO samples listed in Table 7, thymol was the major compound regardless of geographic origin or oil extraction technique. The levels of thymol varied between 18.11% and 55.44%. Other constituents of the thyme EOs are carvacrol, cymene, *γ*-terpinene, (*E*)-caryophyllene, and linalool. The leaves are the main source used for the extraction of thyme EOs, probably because they have the highest yield when compared to branches.

**Bioactivity and bioactive compounds:** Some biological activities of *Thymus* sp. EO are directly related to the chemical composition, which can be influenced by the vegetative stage, seasonal variation of humidity, temperature, harvesting, and post-harvest factors (drying and storage) [199,206].

Recently, a study identified that thyme EO, rich in thymol and carvacrol, could be used for food preservation due to its antibacterial activity against bacterial species, such as *Bacillus cereus*, *Staphylococcus aureus*, *S. epidermidis*, *E. coli*, *Salmonella enteritidis*, and *S. typhimurium* [202]. Similarly, Lemos et al. [199] evaluated thyme EOs against Gram-negative (*S. typhimurium* and *E. coli*) and Gram-positive bacteria (*S. aureus*). The best activities were observed for the samples with the highest levels of thymol and carvacrol. Concerning the antimicrobial activity of thymol, it has been widely described in the literature [202,204], and it has been conjectured that its mechanism of action is involved with the perturbation of the lipid fraction of the plasma membrane, resulting in alterations of its permeability [107].

Regarding the antioxidant activity of some phenolic compounds, a study performed an in vitro assay and SAR analysis to investigate their radical scavenging activity and reported that thymol and its isomer carvacrol inhibit 35.0% and 33.9% of DPPH free radicals, respectively. Additionally, these authors noted that regardless of the position of the alkyl group (ortho, para, or meta), these compounds stabilize the phenoxyl radicals by the inductive effect and thus increase the anti-radical activity; therefore, both thymol and carvacrol and their others position isomers have high antioxidative activities [207].

Thymol and carvacrol have also been reported to have larvicidal and repellent activity against mosquitos [121], and studies have indicated that these monoterpenes act as inhibitors of the odorant-binding proteins of these insects, which are involved with host-seeking recognition [51,208]. Recently, these compounds and *p*-cymene, have been reported to reduce lung emphysema and inflammation [70]. The authors found that these monoterpenes reduced the alveolar enlargement and decreased the levels of cytokines related to inflammation, such as IL-1β, IL-6, IL-8, and IL-17 in bronchoalveolar lavage fluid (BALF), as well as, reduced MMP-9 and p-65-NF-κB-positive cells in the lung parenchyma.

**Antioxidant activity**: It is interesting to apply different methodologies when evaluating the antioxidant activities of EOs. Table 7 displays some recent studies about the antioxidant activity of *T. vulgaris* EOs.

Lemos et al. [199] reported that the major constituents of *T. vulgaris* EOs cultivated in Brazil were thymol (38.99–52.92%), *p*-cymene (14.38–26.58%), and γ-terpinene (10.43–19.09%). They utilized DPPH, ABTS, and FRAP assays to evaluate the antioxidant activities of the EOs, and the results varied according to the method. From DPPH results, the EOs displayed moderate activity. Using the FRAP assay, all samples were classified as strong antioxidants. For the ABTS assay, the EO extracted from the plant material collected in October (2012) showed the best activity, when compared with the synthetic antioxidants BHT and Trolox. They attribute the antioxidant activities to the presence of thymol and carvacrol due to their ability to reduce the free radicals in the three methods investigated. Aprotosoaie et al. [204] also used DPPH, ABTS, and FRAP assays to evaluate thyme EO and reported that the method influenced significantly the antioxidant activity results. The authors also identified that thymol (55.44%), the major constituent in the EO, was responsible for its high antioxidant potential.

The extraction method may cause changes in the chemical composition of the EO. Gedikoğlu et al. [202] subjected thyme to microwave-assisted extraction (MAE) and hydrodistillation (HD). DPPH and FRAP assays were used to evaluate antioxidant activity. The extraction method influenced the FRAP value of thyme EO (MAE = 3.18 ± 0.015 μM Fe^+2^/g; HD = 3.25 ± 0.017 μM Fe^+2^/g). Using DPPH method, MAE (93.77 ± 13.0 μg mL^−1^) provided an EO with higher scavenging activity when compared to HD (159.59 ± 12.79 μg∙mL^−1^), which was associated with the higher thymol levels found in the EO obtained by MAE.

Recently, a study described the antioxidant and antimicrobial efficiency of *T. vulgaris* EO, chitosan, and the mixture of chitosan and the EO (1:1). The EO displayed high radical scavenging activity (DPPH, ABTS, linoleic acid radicals, and FRAP assays). The results confirmed that the thyme EO can be used in commercial applications as an antimicrobial, antioxidant agent, and wound curing agent [205].

## 4. Final Considerations

For centuries, spice plants have formed the basis of traditional medicine and some of their derived substances have been utilized to treat different human diseases. EOs obtained from these plants have also been used as therapeutic agents and have shown supportive uses in remedial practices. EOs are natural sources of bioactive compounds, described by their analgesic, anti-inflammatory, antimicrobial, and cytotoxic activities against cancer cell lines, as well as their antioxidant activities. Recently, a great number of scientific studies have reported new findings related to the bioactivity of some natural compounds present in EOs of well-known spice plants, thus opening up new opportunities for scientific discovery and potential applications in pharmaceutical, food, and cosmetic industries of these natural products. It is important to highlight that despite the fact that some in vitro tests using EOs or their derivative compounds exhibit preliminary data about their biological activities, those studies, when interpreted with the traditional uses of these plants, may shed light on their potential applications and mechanisms of action. Additionally, isolated or synthetic-derivative compounds of plants have been preferred to be applied in pharmaceutical and cosmetic industries due to their predictable and controllable effects in the human body when compared with total content. However, in food industries, the total content of EOs has been investigated as a food preservative due to the presence of antioxidants and antimicrobial compounds, as well as their flavoring properties. Based on that, in the present review, we exhibited and discussed the bioactivity of some chemical components found in these EOs and discussed results related to the antioxidant activity of the total content of EOs and their compounds.

Related to the antioxidant activity of EOs, the natural compounds described with this biological activity represent a relevant fraction of the chemical composition of these natural products. However, in general, these activities of EOs are associated with the synergistic effects resulting from the combination of the constituents present in their chemical composition. It also has been shown that some compounds found in these EOs, such as eugenol, thymol, menthol, eucalyptol, and carvacrol, are mainly responsible for their antioxidant activity. It is also important to highlight the fact that the combination of different in vitro methodologies to evaluate antioxidant activity is necessary to eliminate potential errors and obtain robust results. Finally, biological assays, such as RG inhibition activity and CAA have been pointed out as confirmatory methods to evaluate the effectiveness of these compounds in biological systems and to validate the initial screening of the antioxidant sources.

## Figures and Tables

**Table 1:** Major natural compounds, methodologies applied to determine the antioxidant activity, and the investigated applications of *Cinnamomum zeylanicum* (Lauraceae) essential oils (EOs).

Plant Part	Major Compounds (%)	Antioxidant Activity Assay	Investigated Properties	References
bark	(*E*)-cinnamaldehyde (45.13%), cinnamyl alcohol (8.21%), and eugenol (7.47%).	DPPH β-carotene-linoleic acid	Reported the chemical composition, antioxidant, and antimicrobial activities of the true cinnamon EO.	[85]
barks	(*E*)-cinnamaldehyde (81.39%), (*E*)-cinnamyl acetate (4.20%), and (*Z*)-cinnamaldehyde (3.42%).	PM CUPRAC FRAP DPPH ABTS	Subjected the *C. zeylanicum* EO and its major constituents to different assays: anti-Alzheimer, anti-diabetic, skin whitening, and antioxidant activity.	[91]
barks, leaves	cinnamaldehyde (77.34%), (*E*)-cinnamyl acetate (4.98%), 1,8-cineole (3.19%), 1,4-benzenedicarboxylic acid (3.55%)	PM DPPH hydrogen peroxide radical scavenging assay	Evaluated the antioxidant activity and antiproliferative effect against cancer cells lines (HeLa and Raji)	[83]

**Table 2:** Major natural compounds, methodologies applied to determine the antioxidant activity, and the investigated applications of *Mentha piperita* (Lamiaceae) EOs.

Plant Part	Major Compounds (%)	Antioxidant Activity Assay	Investigated Properties	References
leaves	menthol (38.45%), menthone (21.8%), eucalyptol (5.62%), and neo-menthol (4.19%).	DPPH TBARS CAA Reducing power cellular-based antioxidant activity	Evaluated the chemical composition and antioxidant properties using different methods.	[37]
leaves	menthol (30.69%), menthone (14.51%), menthyl acetate (12.86%), and *neo*-menthol (9.26%)	DPPH Hydroxyl radical scavenging activity	Investigated the chemical composition, anti-inflammatory, and cytotoxic (against different human cancer cell lines) activities of EO from leaves of *M. piperita* collected in China	[57]
leaves	linalool (51.8%), epoxyocimene (19.3%), and sesquiphellandrene (9.4%).	DPPH	Evaluated the antioxidant, cytotoxic (*Artemia salina*), antibacterial (*Staphylococcus aureus and Escherichia coli*), and larvicidal (*Aedes aegypti*) activities.	[109]
leaves	menthol (32.93%), menthone (24.41%), *cis*-caran (8.08%), and eucalyptol (1,8-cineole, 7.89%)	DPPH	Investigated the chemical composition, the antioxidant and antimicrobial activity of *M. piperita* EO against bacterial strains and *Candida albicans*	[53]

**Table 3:** Major natural compounds, methodologies applied to determine the antioxidant activity, and the investigated applications of *Ocimum basilicum* (Lamiaceae) EOs.

Plant Part	Major Compounds (%)	Antioxidant Activity Assay	Investigated Properties	References
leaves and stems	linalool (27.64–31.66%), methyl chavicol (15.96–17.37%), methyl cinnamate (10.48–15.14%), bicyclo-sesquiphellandrene (6.01–7.01%), and eugenol (2.79–7.34%)	DPPH	Evaluated the chemical composition and antioxidant activity of the EOs obtained from three locations of Egypt (Assiut, Beni Suef, and Minia). They also described the antioxidant properties of sweet basil ethanolic extracts.	[115]
leaves	methyl chavicol (75.10%), eucalyptol (4.60%), and linalool (4.50%)	DPPH	Demonstrated the usefulness of the simplex-lattice mixture design method to optimize the antioxidant profile of an EO mixture (basil, marjoram, and rosemary).	[126]
leaves	*trans*-β-guaiene (16.89%), α-selinene (15.66%), phytol (11.68%), 9-methoxybicyclo[6.1.0]nona-2,4,6-triene (11.36%), and longifolenealdehyde (8.74%).	DPPH Hydroxyl radical scavenging activity Superoxide scavenging activity FRAP	Evaluated the pure *O. basilicum* EO and its encapsulated form (utilizing Polysorbate 80). Both were described by its antibacterial, antioxidant, and larvicidal (*Culex quinquefasciatus*) activities.	[119]
leaves	methyl chavicol (41.40%), 1,6-octadien-3-ol, 3,7-dimethyl (29.49%), and trans-α-bergamotene (5.32%)	TBARS	Reported the preservative effect of basil EO on physicochemical characteristics and lipid oxidation of minced beef during storage.	[28]
leaves	linalool (22.45–29.41%), camphor (13.70–16.00%), eugenol (11.39–19.54%), eucalyptol (9.06–22.21%), and germacrene D (6.02–6,98%).	ORAC	Described the oxygen introduction during the hydrodistillation and the effect of this modification on the antioxidant activity of basil EO.	[129]
leaves	linalool (19–57.2%), methyl chavicol (4.5–59.5%), *E*-methyl cinnamate (45.9%), and methyl eugenol (0.1–74.7%)	ABTS FRAP	They reported the chemical compositions, antioxidant properties, and antimicrobial activities of different *O. basilicum* varieties.	[125]
leaves	linalool (23.32–28.10%), methyl chavicol (15.74–20.64%), and methyl cinnamate (19.31–20.20%).	ORAC	Evaluated the effect of steam distillation and hydrodistillation extraction methods on the yield, composition, and antioxidant activity of basil EO.	[128]
leaves	linalool (31.60%), methyl chavicol (23.80%), β-elemene (6.90%), γ-muurolene (4.10%), and α-guiaene (2.70%).	DPPH	Displayed the antimicrobial, antioxidant activity, and chemical composition of basil EO.	[127]
leaves	eugenol (15.2924.88%), eucalyptol (16.68–20.34%), methyl eugenol (10.84–40.69%), linalool (4.98–20.88%), and α-humuleno (4.53–9.53%)	DPPH ABTS *β*-carotene-linoleic acid	Evaluated the effect of elicitation with jasmonic acid on the plant yield and composition of basil EOs. They also described the antioxidant and anti-inflammatory activities of the EOs.	[130]

**Table 4:** Major natural compounds, methodologies applied to determine the antioxidant activity, and the investigated applications of *Origanum vulgare* (Lamiaceae) EOs.

Plant Part	Major Compounds (%)	Antioxidant Activity Assay	Investigated Properties	References
aerial parts	*O. vulgare vulgare:* thymol (58.31%), carvacrol (16.11%), and *p*-cymene (13.45%)	DPPH ABTS CUPRAC FRAP	Evaluated the antimicrobial activity against bacterial species, chemical composition, antioxidant, and inhibitory enzymatic activities of *O. vulgare* EO.	[151]
aerial parts	*O. vulgare hirtum:* linalool (96.31%), β-caryophyllene (1.27%), and carvacrol (0.33%)	DPPH ABTS CUPRAC FRAP	Evaluated the antimicrobial activity against bacterial species, chemical composition, antioxidant, and inhibitory enzymatic activities of *O. vulgare* EO.	[151]
aerial parts	thymol (37.12%), *γ*-terpinene (9.66%), carvacrol (9.57%), and *cis*-α-bisabolene (6.80%)	DPPH FRAP	Evaluated the chemical composition and the antioxidant activity of oregano EO.	[154]
leaves	*p*-cymene (35.7–46.3%), *γ*-terpinene (11.7–24.2%), and thymol (18.4–39.1%)	DPPH	Investigated the variation of the chemical composition of *O. vulgare* L. subsp. *glandulosum* EO collected from three localities of north Tunisia and their antioxidant activities.	[143]
aerial parts	thymol (45%) and carvacrol (37.4%)	DPPH TBARS	Reported the chemical composition and the antioxidant activity of *O. vulgare* EO.	[155]
leaves, flowers, and branches	carvacrol (45.09–46.71%), thymol (14,67–15.72%), and *p*-cymene (5.15–5.80%).	DPPH	Evaluated the effect of different drying methods on the EO yield, chemical composition, and antioxidant activity.	[152]
aerial parts	linalool (4.64–12.50%), caryophyllene oxide (5.00–10.40%), (*E*)-caryophyllene (3.40–26.41%), germacrene-D (0.00–10.46%), bornyl acetate (0.00–10.40%), thymol (0.00–60.30%), and myrceno (0.00–8.64%)	DPPH FRAP	Investigated the variation in the chemical composition, yield and in vitro antioxidant activity of *O. vulgare* EO.	[153]

**Table 5:** Major natural compounds, methodologies applied to determine the antioxidant activity, and the investigated applications of *Piper nigrum* (Piperaceae) EOs.

Plant Part	Major Compounds (%)	Antioxidant Activity Assay	Investigated Properties	References
Fruit	(*E*)-caryophyllene (51.12%), β-thujene (20.58%), and β-selinene (5.59%)	DPPH ABTS	Evaluated the chemical composition, antihyperuricemic, antioxidant, and herbicidal activities of *P. nigrum* EO. Black pepper EO has been effective in controlling invasive and problematic weeds in agricultural practice.	[157]
fruit	caryophyllene (25.58–62.23%), (1R)-2,6,6-trimethylbicyclo[3.1.1]hept-2-ene (8.71–40.85%), 3-carene (6.20–26.84%), and D-limonene (4.39–23.66%)	DPPH ABTS FRAP Scavenging superoxide anion free radical activity	Evaluated the chemical composition, antioxidant, and antifungal activities of *P. nigrum* EO from different regions.	[160]
fruit	(*E*)-caryophyllene	DPPH	Characterized the encapsulated black pepper EO in hydroxypropyl-β-cyclodextrin and described its antioxidant, and antibacterial activities.	[169]
fruit	caryophyllene (23.98%), limonene (14.36%), and α-terpinene (13.26%)	Superoxide radical scavenging Hydroxyl radical scavenging TBARS DPPH FRAP	Evaluated the antinociceptive, antioxidant, and anti-inflammatory activities of *P. nigrum* EO.	[170]
fruit	(*E*)-caryophyllene (15.64–25.38%), limonene (14.95–15.64%), sabinene (13.19–13.63), and 3-carene (8.56–9.34%)	DPPH	Optimized the supercritical carbon dioxide process to improve the antioxidant activity of *P. nigrum*.	[171]

**Table 6:** Major natural compounds, methodologies applied to determine the antioxidant activity, and the investigated applications of *Rosmarinus officinalis* (Lamiaceae) EOs.

Plant Organ	Major Compounds (%)	Antioxidant Activity Assay	Investigated Properties	References
leaves	eucalyptol (15.2%), c amphor (15.1%), and β-pinene (11.0%)	DPPH	Demonstrated the usefulness of the simplex-lattice mixture design method to optimize the antioxidant profile of an EO mixture (basil, marjoram, and rosemary).	[126]
leaves	α-pinene (14.69–20.81%), eucalyptol (5.63–26.89%), and camphor (4.02–24.82%)	DPPH	Reported the antioxidant, antibacterial and chemical composition of rosemary EO extracted from plants collected in different regions.	[48]
aerial parts	eucalyptol (23.67%), camphor (18.74%), and borneol (15.46%)	DPPH FRAP	Evaluated the chemical composition, antileishmanial, antibacterial, and antioxidant activities of rosemary EO.	[189]
Leaves	γ-cadinene (29.93%), camphor (14.76–28.42%), and eucalyptol (10.60–15.36%)	ABTS	Used three different extraction methods (hydrodistillation, steam distillation, and supercritical CO_2_) and evaluated their chemical profile and antioxidant activity.	[190]
Leaves	eucalyptol (35.15–50.28%), camphor (12.71–13.08%), α-pinene (5.60–13.92%), borneol (7.42–9.81%), and α-terpineol (1.76–15.40%)	DPPH	Described a method of rapeseed oil aromatization with rosemary. The EO aromatized was evaluated about the chemical composition and antioxidant activity.	[5]
Leaves	eucalyptol (11.33–37.29%), camphor (8.81–40.35%), and α-pinene (2.60–28.68%),	DPPH	Evaluated the antioxidant activity of rosemary EO fractions obtained by molecular distillation and their effect on oxidative stability of sunflower oil.	[188]
aerial parts	camphor (14.80–42.50%), eucalyptol (8.00–26.40%), α-pinene (4.10–13.20%), and myrcene (1.00–30.30%)	DPPH FRAP	Extracted the EO from several cultivars of rosemary and evaluated their antioxidant activity.	[178]
aerial part	eucalyptol (35.32%), (*E*)-caryophyllene (14.47%), borneol (9.37%), camphor (8.97%), α-pinene (7.90%), and α-thujone (6.42%)	DPPH CAT SOD	Evaluated the potential of rosemary EO as anti-hyperlipidemic, anti-hyperglycemic, and antioxidant.	[191]
Leaves	eucalyptol (42.86–46.76%), camphor (16.26–23.42%), α-pinene (6.37–9.19%), camphene (2.27–4.37%), and borneol (4.00–4.33%)	DPPH FRAP TBARS	Compared the chemical profile and antioxidant activity of the EOs of *R. officinalis* collected from China and Iran regions.	[192]

**Table 7:** Major natural compounds, methodologies applied to determine the antioxidant activity, and the investigated applications of *Thymus vulgaris* (Lamiaceae) EOs.

Plant Part	Major Compounds (%)	Antioxidant Activity Assay	Investigated Properties	References
leaves	thymol (41.04%), eucalyptol (14.26%), γ-terpinene (12.06%), *p-c*ymene (10.50%), α-terpinene (9.22%), linalool (2.80%), and carvacrol (2.77%)	DPPH ABTS β-Carotene-linoleic acid FRAP	Assessed the antioxidant and antimicrobial efficiency of the thyme EO isolated and combined with chitosan (mixture 1:1). Reported the chemical composition of the EO.	[205]
aerial parts	thymol (55.44%), *m*-cymene (11.88%), γ-terpinene (5.74%), and *o*-cymen-5-ol (5.14%)	DPPH ABTS FRAP	Evaluated the chemical composition, antioxidant properties, and antifungal activities against *Aspergillus flavus* of the EO obtained from two cultivars of *T. vulgaris*.	[204]
whole plant	thymol (50.53–55.30%), *p*-cymene (11.20–11.79%), carvacrol (6.65–8.70%), and (*E*)-caryophyllene (4.20–4.88%)	DPPH FRAP	Investigated two different techniques to extract the EO (hydrodistillation and microwave-assisted extraction) to evaluate possible changes in oil yield (%), chemical composition, antioxidant, and antimicrobial activities. They also described the extract’s compositions and activities.	[202]
leaves	thymol (25.78%), carvacrol (17.47%), thymoquinone (7.11%), eugenol (6.36%), β-pinene (6.31%), β-ocymene (5.80%), and isophytol (5.70%)	DPPH PM	Evaluated the chemical composition, total phenolic, flavonoids, antioxidant, antibacterial and cytotoxic activities of the EO from *T. vulgaris* cultivated in Iran.	[196]
aerial parts	thymol (18.11–35.00%), *p*-cymene (6.61–25.20%), (*E*)-caryophyllene (5.38–8.47%), thymyl methyl ether (4.28–8.44%), linalool (3.72–4.66%), and γ-terpinene (2.23–6.04%)	DPPH	Reported the use of spraying salicylic acid on drought-stressed plants and its influences on the EO yields, chemical composition, antioxidant, and polyphenolic content.	[201]
leaves and branches	thymol (38.99–52.92%), *o*-cymene (14.38–26.58%), γ-terpinene (10.43–19.90%), and linalool (2.39–3.56%)	DPPH ABTS FRAP	Evaluated the seasonal influences on the composition, antibacterial, and antioxidant activities of *T. vulgaris* EO.	[199]
leaves	thymol (40.02%), carvacrol (18.31%), *p*-cymene (16.78%), linalool (4.84%), and γ-terpinen-7-al (4.16%)	DPPH Potassium ferricyanide CUPRAC	Reported the chemical composition and antioxidant activity of thyme EO.	[209]
leaves	thymol (39.79%), cymene (17.33%), γ-terpinene (13.45%), α-pinene (5.02%), (*E*)-caryophyllene (4.17%), and linalool (3.87%)	DPPH	Analyzed the EO chemical composition and the antimicrobial activities of a specimen of *T. vulgaris* from Vietnam.	[210]

## Abbreviations

ABTS	2:2’-azino-bis (3-ethylbenzothiazoline-6-sulfonic acid)
AAPH	2,2-*a*zobis (2-methylpropionamidine) hydrochloride
BHT	butylhydroxytoluene
CUPRAC	cupric ion reducing antioxidant capacity assay
DMSO	dimethylsulfoxide
DCFH_2_	dihydrodichlorofluorescein
DCF	dichlorofluorescein
DPPH	1,1-diphenyl-2-picrylhydrazyl
EO	essential oil
FRAP	ferric antioxidant power reduction assay
GR	glutathione reductase
GSH	reduced glutathione
GSSG	oxidized glutathione
MDA	malondialdehyde
NBT	nitro-blue tetrazolium
ORAC	oxygen radical absorption capacity assay
PM	phosphomolybdenum assay
ROS	reactive oxygen species
TBARS	thiobarbituric acid reactive substances assay
TEAC	Trolox equivalent antioxidant activity
TPTZ	2,4,6-tripyridyl-s-triazine
UV-Vis	ultraviolet-visible
